# Translating Ethics into Practice: Providing Long-Term Cardiometabolic and Cardiovascular Disease Care for Research Participants in Africa

**DOI:** 10.5334/gh.1206

**Published:** 2023-06-16

**Authors:** Cody Cichowitz, Godfrey Kisigo, Grace Ruselu, Bahati Wajanga, Bernard Desderius, Anthony O. Etyang, Saidi Kapiga, Robert Peck

**Affiliations:** 1 Division of Cardiology, University of California San Francisco, San Francisco, CA, USA; 2 Department of Medicine, Center for Global Health, Massachusetts General Hospital, Boston, MA, USA; 3 Mwanza Intervention Trials Unit, National Institute for Medical Research, Mwanza, Tanzania; 4 Bugando Medical Centre, Mwanza, Tanzania; 5 Department of Infectious Disease Epidemiology, London School of Hygiene and Tropical Medicine, London, UK; 6 KEMRI-Wellcome Trust Research Programme, Kilifi, Kenya; 7 Center for Global Health, Weill Cornell Medicine, New York, USA

**Keywords:** Global Cardiology, Non-communicable diseases, Ethics, Equity

In sub-Saharan Africa (SSA), health systems face significant challenges responding to the growing burden of cardiometabolic and cardiovascular diseases (CVDs) [1,2], and most adults in SSA lack access to primary care [3]. Consequently, many research participants will be diagnosed with untreated cardiometabolic and cardiovascular disease during internationally funded research. In 2019, we argued that there is an ethical imperative for researchers in SSA and other low-income settings to facilitate the ‘highest achievable standard of care’ for all untreated non-communicable diseases identified during research [4]. However, no standards exist for researchers to implement this ethical imperative and facilitate long-term access to treatment.

Over the past four years we have been working to ensure long-term access to cardiometabolic care for participants in our own ongoing CVD cohort in Tanzania [5,6,7]. Prior to study initiation, we committed to provide free access to treatment for newly diagnosed hypertension, diabetes, coronary artery disease, and heart failure. Our group’s experience illustrates many opportunities and challenges associated with securing long-term cardiometabolic and CVD treatment during research.

**Figure 1:**
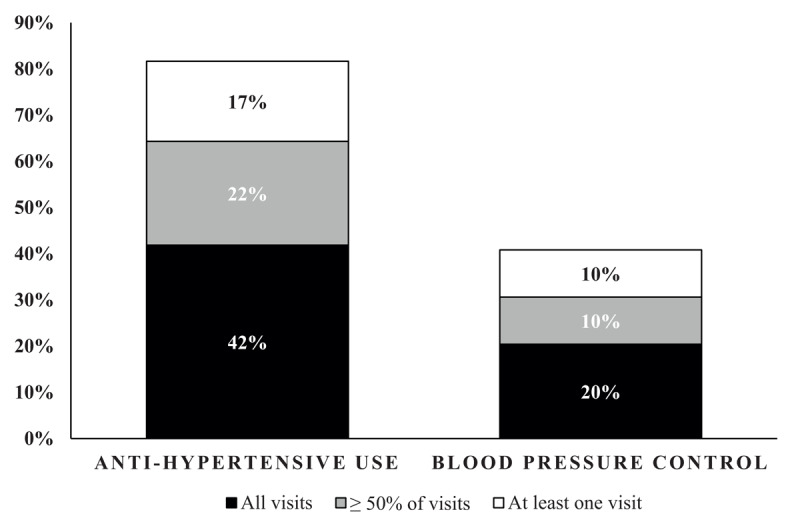
Hypertension outcomes during follow-up visits after provision of free medications in a cohort study (n = 98). Hypertension was defined as a final blood pressure > 140/90. Three successive, unobserved blood pressure measurements were obtained on the right arm with a 60-second interval between measurements. Average SBP/DBP was calculated using the second and third measurements. All participants diagnosed with hypertension were offered free medications at every study visit. Medication use and disease control was assessed at every subsequent visit following a diagnosis. Medication use was assessed through administered surveys, during which participants reported medication use during the week prior to the study visit. Blood pressure control was defined as a blood pressure ≤ 140/90.

In our cohort of 1,000 adults who have been engaged in an observational study for the last seven years, 180 (18%) of participants have been diagnosed with a new non-communicable disease, including hypertension (n = 98), diabetes (n = 61), and CVD (n = 26). As demonstrated in Figure 1, our outcomes for hypertension treatment and control are much better those reported in the Tanzania community [8,9], but still more work is needed. For participants diagnosed with diabetes, medication uptake (7 of 61) and glycemic control (5 of 61) has been unacceptably low. All participants with symptomatic CVD adhered to recommended care, yet we often struggled to link participants to long-term subspeciality care.

Learning from these data, we have identified several barriers to ensuring long-term access to cardiometabolic and CVD care and have implemented several solutions. We recently hired a medical doctor who connects participants with treating clinicians and ensures guideline directed therapy is offered. We also now provide health insurance to participants diagnosed with CVD to improve linkage to subspeciality care. We have created feedback systems so that treating clinicians are aware of participants with cardiometabolic or cardiovascular disease who have not initiated medication, are non-adherent, or have not achieved disease control. In collaboration with treating clinicians, we have also developed standardized educational information, referral pathways, and treatment algorithms. Together with our partners in health system leadership, we believe that these efforts can help to improve CVD care beyond our own study.

The annual costs associated with diagnostics, medications, insurance, and study team effort amount to ~$10,000 USD, which are paid for from private funds. While not insignificant, these costs are nominal compared to our annual NIH funding of over $500,000 USD.

We acknowledge that research should remain independent from clinical care, yet we maintain that the limited availability of CVD care in SSA necessitates that researchers actively work to secure basic health care and human rights for participants. We must partner with local health systems to foster sustainability of treatment. In solidarity with many HIV researchers who came before us and helped ensure immediate access to ART prior to widespread availability, we believe that the imperative to provide lifesaving treatment requires immediate action and advocacy.

Researchers have a moral obligation to ensure that research participants have durable access to the highest possible local standard of care for cardiometabolic disease, CVD, and other untreated non-communicable diseases. Funding agencies should allow for the purchasing of medication and health insurance and evaluate research based upon plans to address cardiovascular health during and after project implementation. Journals should require investigators to describe access to non-communicable disease care for participants. Finally, researchers should engage community stakeholders to advocate for high-quality CVD and cardiometabolic care. These changes are essential to fostering equity in global health research and maximizing the impact of scientific inquiry in Africa.
